# Significantly Elevated *FMR1* mRNA and Mosaicism for Methylated Premutation and Full Mutation Alleles in Two Brothers with Autism Features Referred for Fragile X Testing

**DOI:** 10.3390/ijms20163907

**Published:** 2019-08-11

**Authors:** Michael Field, Tracy Dudding-Byth, Marta Arpone, Emma K. Baker, Solange M. Aliaga, Carolyn Rogers, Chriselle Hickerton, David Francis, Dean G. Phelan, Elizabeth E. Palmer, David J. Amor, Howard Slater, Lesley Bretherton, Ling Ling, David E. Godler

**Affiliations:** 1 Genetics of Learning Disability Service, Hunter Genetics, Waratah, NSW 2298, Australia; 2 Grow-up Well Priority Research Centre, University of Newcastle, Newcastle, NSW 2308, Australia; 3 Diagnosis and Development, Murdoch Children’s Research Institute, Royal Children’s Hospital, Parkville, VIC 3052, Australia; 4 Department of Paediatrics, Faculty of Medicine, Dentistry and Health Sciences, University of Melbourne, Parkville, VIC 3052, Australia; 5 School of Psychology and Public Health, La Trobe University, Bundoora, VIC 3086, Australia; 6 Victorian Clinical Genetics Services, Murdoch Children’s Research Institute, Royal Children’s Hospital, Parkville, VIC 3052, Australia; 7 Psychology Service, The Royal Children’s Hospital, Parkville, VIC 3052, Australia; 8 Melbourne School of Psychological Sciences, University of Melbourne, Parkville, VIC 3052, Australia

**Keywords:** fragile X syndrome, autism, RNA toxicity, DNA methylation, mosaicism, pediatrics, MS-QMA, AmplideX

## Abstract

Although fragile X syndrome (FXS) is caused by a hypermethylated full mutation (FM) expansion with ≥200 cytosine-guanine-guanine (CGG) repeats, and a decrease in *FMR1* mRNA and its protein (FMRP), incomplete silencing has been associated with more severe autism features in FXS males. This study reports on brothers (B1 and B2), aged 5 and 2 years, with autistic features and language delay, but a higher non-verbal IQ in comparison to typical FXS. CGG sizing using AmplideX PCR only identified premutation (PM: 55–199 CGGs) alleles in blood. Similarly, follow-up in B1 only revealed PM alleles in saliva and skin fibroblasts; whereas, an FM expansion was detected in both saliva and buccal DNA of B2. While Southern blot analysis of blood detected an unmethylated FM, methylation analysis with a more sensitive methodology showed that B1 had partially methylated PM alleles in blood and fibroblasts, which were completely unmethylated in buccal and saliva cells. In contrast, B2 was partially methylated in all tested tissues. Moreover, both brothers had *FMR1* mRNA ~5 fold higher values than those of controls, FXS and PM cohorts. In conclusion, the presence of unmethylated FM and/or PM in both brothers may lead to an overexpression of toxic expanded mRNA in some cells, which may contribute to neurodevelopmental problems, including elevated autism features.

## 1. Introduction

Fragile X syndrome (FXS) is a leading single-gene cause of inherited intellectual disability (ID), with a prevalence of up to 1 in 4000 [1]. The vast majority (~90%) of males affected by FXS, and ~50% of females, show autism spectrum disorder (ASD) features, including speech perseveration, compulsions, echolalia, repetitive behaviors, poor eye contact, and deficits in social communication [2,3,4,5]. Many of these features are shared with idiopathic ASD [6]. The primary molecular cause of FXS is abnormal regulation of the fragile X mental retardation 1 (*FMR1*) gene due to the presence of ≥200 cytosine-guanine-guanine (CGG) repeats, termed full mutation (FM), within the *FMR1* promoter (reviewed in Kraan et al. [7]). These FM alleles are thought to silence *FMR1* transcription [8,9,10,11,12] through epigenetic changes at the *FMR1* promoter, including increased DNA methylation. Loss of *FMR1* mRNA in turn leads to depletion of the fragile X mental retardation protein (FMRP) [13,14,15,16], which is the primary cause of FXS symptomology, as FMRP has a critical function in synaptic plasticity and normal brain development [17,18,19,20,21].

In contrast, smaller CGG alleles (55–199 repeats), termed premutation (PM), usually have an unmethylated *FMR1* promoter, but abnormally increased levels of expanded PM mRNA [22,23]. In some PM carriers, the transcription of *FMR1* mRNA from expanded alleles has been associated with “RNA gain of function” toxicity, implicated in late onset disorders such as Fragile X-associated Tremor/Ataxia Syndrome (FXTAS) [22,23]. Other factors proposed to contribute to PM-related disorders are expanded repeat associated non-AUG translation, and increased transcription of *ASFMR1/FMR4* originating from the same locus as *FMR1* (reviewed in Kraan et al. [7]). In FXS, these mechanisms have not been comprehensively studied, but may explain significant variability in the type and severity of the FXS phenotype, beyond FMRP deficiency. This is consistent with our recent study suggesting that, in the majority (60%) of FXS males, FM mRNA can still be detected, and that this incomplete silencing in males <19 years of age is associated with more severe autism features, but not more severe ID [6]. This led us to propose that FXS can be stratified based on complete and incomplete silencing, with the two reciprocal mechanisms of RNA toxicity and FMRP deficiency contributing to overlapping aspects of FXS, namely the ID and autism phenotype in each individual that has incomplete silencing. This is particularly important as mosaicism for active and inactive expanded alleles may be more common than previously reported (10% to 40%) [5,11,15,24,25,26,27,28,29], based on the detection of *FMR1* mRNA in more than 60% of FM males [6]. The difference of >20% in the reported prevalence of mosaicism in FXS by Southern blot analysis as opposed to incomplete *FMR1* silencing detected by real-time PCR [6], is likely due to the limited analytical sensitivity of methylation-sensitive Southern blot analysis (the ‘gold standard’ FXS diagnostic test used in earlier studies) that does not detect mosaic alleles in less than 20% of cells [26]. Extreme examples of low level methylation mosaicism found in <20% of cells have previously been described in rare adult individuals with unmethylated FM (UFM) alleles that typically have mild ID (but are at risk of premutation phenotypes such as FXTAS) [30]. 

This study, for the first time, describes two brothers with UFM alleles detected by Southern blot analysis in a pediatric setting. Based on non-verbal intellectual functioning assessments, the brothers are ‘higher functioning’ compared to typical FXS, but have autism features ranging from mild to severe. The detailed clinical and molecular follow-up is described. This includes the detection of low-level mosaicism for methylated PM and FM alleles in different tissues. The CGG sizing and *FMR1* mRNA analysis of the two brothers are also compared with family members ascertained through cascade testing, typical FXS, age-matched children with PM, and typically developing (TD) controls.

## 2. Results 

### 2.1. Medical History 

**Figure 1:**
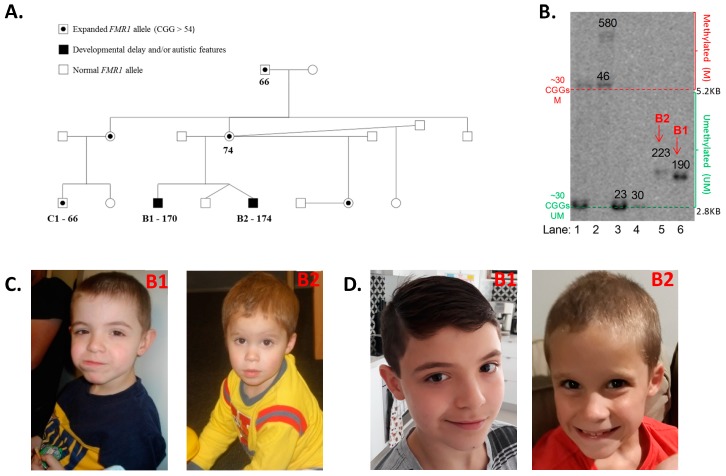
(**A**) Family pedigree, with expansion sizes from AmplideX testing. None of the individuals had manifested symptoms consistent with fragile X syndrome or fragile X-associated disorders, such as Fragile X-associated Tremor/Ataxia Syndrome (FXTAS) or Fragile X-associated Primary Ovarian Insufficiency (FXPOI). The brothers’ have two half-sisters, one of which was identified as carrying a premutation (PM) allele and the other as carrying a normal fragile X mental retardation 1 (*FMR1*) allele. B1′s cousin (C1) was identified at 1 year and 11 months of age as carrying an *FMR1* PM allele of 66 CGG repeats. (**B**) Methylation-sensitive Southern blot analysis of the *NruI* restriction site within the *FMR1* CpG island in blood. The DNA samples from the two brothers in question are located in lanes 5 and 6. B2 has a 223 CGG unmethylated allele, while B1 has a 190 CGG repeat unmethylated allele. A typical female (CGG < 44) is in lane 1, while typical males (CGG < 44) are in lanes 3 and 4; and a full mutation (FM) female with a 100% methylated 580 CGG allele is in lane 2. The numbers superimposed on the blot indicate CGG sizing. The lower limit of detection for the Southern blot analysis of FM alleles on a normal allele background is 20%. (**C**) Photos of B1 and B2 at the ages of 5 and 2 years, respectively; written informed consent was obtained from the mother of the brothers to include these images in the publication (**D**) Photos of B1 and B2 at the ages of 10 and 7 years, respectively; written informed consent was obtained from the mother of the brothers to include these images in the publication. Note: Comparator DNA used of methylation-sensitive Southern blot analysis in (**B**) were also sized using standard CGG sizing PCR.

B1 was born at term after an uncomplicated pregnancy and delivery. His parents became concerned with his development from an early age. He had extreme shyness, disliked new social circumstances, avoided some physical contact, and had limited eye contact. He was difficult to settle and he was often irritable and upset. His early motor milestones were normal, but language was significantly delayed. A clinical evaluation with the Griffiths Mental Developmental Scales, Extended Revised led to a diagnosis of mild global developmental delay, with significant difficulties with language and play skills. Fragile X testing by Southern blot analysis of DNA extracted from blood and microarray analysis were performed at the age of 3 years. Southern blot analysis detected an abnormal 1.42 kb pfxa3 fragment equivalent to approximately 170 CGG repeats expansion. No pathogenic copy number variants (ISCA 60K) were identified through a chromosomal microarray. The results of psychometric testing (Stanford-Binet Intelligence Scales 5th Edition) [31] at the age of 4.5 years were consistent with a mild ID, with relative strengths in nonverbal abilities. The Adaptive Behavioural Assessment System (ABAS-II) results indicated a mild deficit in everyday functioning. He was treated with fluoxetine, which improved his social anxiety. After B1’s fragile X genetic testing, cascade testing was carried out for other family members. The mother of both brothers was found to have a 74 CGG expanded allele; the maternal half-sister, maternal aunt, and maternal grandfather were also all identified as having a PM allele (Figure 1A). 

B2 is the younger brother of B1, who is a dizygous twin. The twins were conceived before the *FMR1* CGG expansion was identified in B1. B2 and his twin brother were delivered at term by elective caesarean section, in good condition after a pregnancy complicated by gestational hypertension. The twins were admitted for two days in the newborn nursery for blood sugar instability and required intravenous fluids. However, there were no ongoing neonatal concerns and the boys were discharged home on day four of life. There were no concerns about B2′s development in the first year of life; he fed and slept well and all his motor milestones were in the normal range. He had surgery for left cryptorchidism, but his medical history was otherwise unremarkable. B2′s mother became concerned about his development and behaviour around two years of age. Although B2 did not have typical craniofacial features of fragile X syndrome, he had a slightly longer face than his twin brother, which was visible at the time of assessment (Figure 1C) and also more recently (Figure 1D). He was normocephalic, had a normal tone and power, with a normal gait, and did not have joint hypermobility. At 9 months of age, fragile X PCR with AmplideX commercial kit was performed on their DNA extracted from blood, which identified a normal triplet repeat size (29 CGG repeats) in one of the twins, but a 174 PM CGG repeats expansion in B2. 

### 2.2. Extended FMR1 CGG Testing

Extended *FMR1* testing was initiated to test the hypothesis that the neurodevelopmental phenotypes in B1 and B2 were due to the presence of FM alleles in tissues other than blood. For these extended studies, B1 and B2 were recruited into the FREE FX study at 5.6 and 2.7 years of age, respectively, but were not included in previous FREE FX study publications due to uncertainty about molecular diagnosis [6]. 

**Figure 2:**
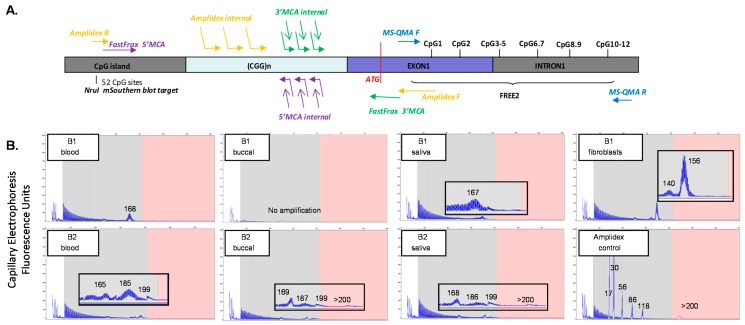
(**A**) Organization of the fragile X mental retardation 1 (*FMR1*) 5′ region, including the cytosine-guanine-guanine (CGG) expansion and primer binding site locations for AmplideX CGG sizing PCR, FastFraX High-Resolution Melt Analysis, and Methylation-Specific Quantitative Melt Analysis (MS-QMA), in relation to Fragile X-Related Epigenetic Element 2 (FREE2); the *FMR1* CpG island and methylation sensitive restriction site *NruI* analysed using routine fragile X Southern blot testing; and two *HpaII* sites targeted by AmplideX methylation PCR. Note: The *FMR1* translation start site is indicated in red overlapping with FastFraX 3′ melting curve analysis (MCA) primer. (**B**) AmplideX triplet-repeat primed long range PCR results for B1 and B2, for blood, buccal, saliva, and fibroblast DNA. Note: a reference sample co-run with the samples in question is included as the AmplideX sizing control in (**B**).

In blood, methylation-sensitive Southern blot analysis demonstrated the presence of unmethylated alleles of 190 CGGs in B1 and 223 CGGs in B2 (Figure 1B). These results are consistent with those previously reported for rare ‘high functioning’ FXS males with fully unmethylated FM alleles [32,33,34]. AmplideX testing of blood at the time of the FREE FX study assessments showed an allele of 168 CGGs in B1, while for B2, there were three alleles detected of 165, 185, and 199 CGGs (Figure 2B). AmplideX CGG sizing analysis in tissues other than blood showed that for B1, CGG sizing in saliva was consistent with that in blood. However, in fibroblasts, the sizing was different, with mosaicism for 140 CGG and 156 CGG alleles detected. In contrast, for B2, all alleles in the PM range were consistent between the tissues tested. However, for B2, in buccal epithelial cells (BEC) and saliva, FM alleles were also detected, which were not identified in blood. Subsequently, B2 received a molecular diagnosis of FXS that was not initially given by standard testing of blood. 

The variability in CGG sizing between tissues was also investigated in both brothers using FastFraX 5′ and 3′ melting curve analysis (MCA) assays, using samples from C1 (cousin, as reference) [35]. Surprisingly, there was no amplification by the 3′ MCA assay in B1 and B2, while amplification occurred by 3′ MCA for C1, with the 5′ MCA performed on the same samples from B1, B2, and C1 displaying amplification, as expected (Supplemental Figure S1). This suggested that one of the primer sites for the 3′ MCA assay had a de novo sequence change of unknown significance in all tissues tested in both brothers that prevented primer binding and amplification, but not in their cousin. As a follow-up, sanger sequencing of the 3′ MCA *FMR1* exon 1 binding site inclusive of the ATG translation start site, and surrounding regions, was performed. However, no sequence variants were detected in both brothers compared to controls (Supplemental Figures S3 and S4). This suggested that a de novo sequence change was present for the binding site of the primer anchored at the 3′ end of the CGG repeat, rather than the primer overlapping with the ATG site within the exon 1.

### 2.3. FMR1 Methylation and Gene Expression Analyses 

**Figure 3:**
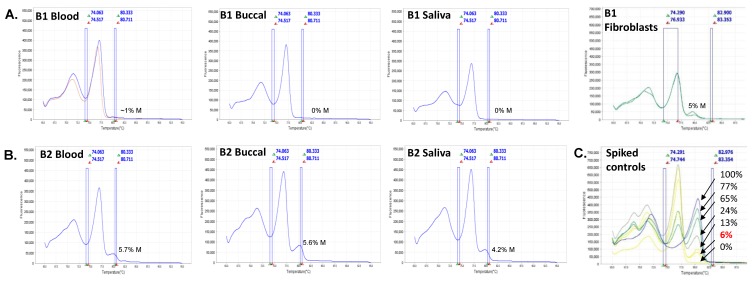
Derivative curve high-resolution melt profiles and mean methylation output ratios of CpG sites located within the Fragile X-Related Epigenetic Element 2 (FREE2) region assessed using Methylation-Specific Quantitative Melt Analysis (MS-QMA) between different tissues for (**A**) B1 and (**B**) B2. (**C**) DNA samples from lymphoblasts of a fragile X syndrome (FXS) male with the fragile X mental retardation 1 (*FMR1*) promoter 100% methylated spiked with DNA from a typically developing control (cytosine-guanine-guanine (CGG) < 44) with the *FMR1* promoter 0% methylated. These samples were mixed at different ratios for the MS-QMA methylation reference curve, with the expected % of methylation indicated on the plot for each derivative curve profile.

**Figure 4:**
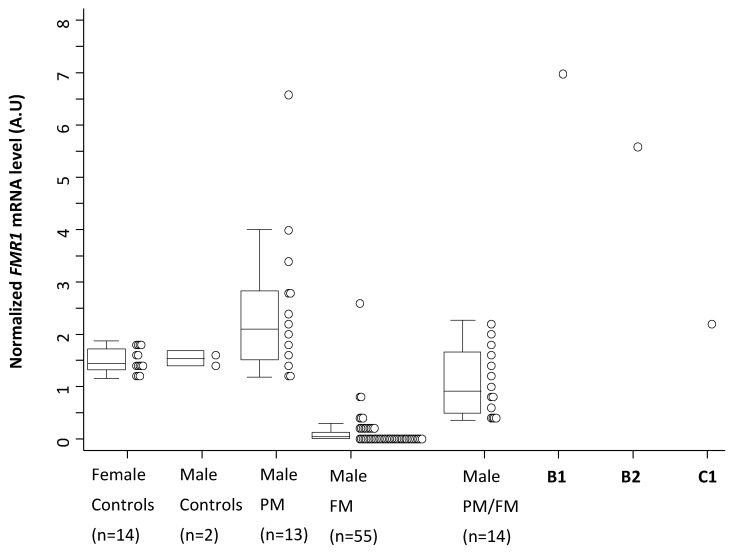
Comparison of fragile X mental retardation 1 (*FMR1*) mRNA levels in blood in the probands, control, premutation (PM), full mutation (FM) only, and PM/FM mosaic reference cohorts. Note: Control reference ranges included in blood of females (age 22 to 54 years; cytosine-guanine-guanine (CGG) < 44) and males (age 7.7–8.1 years) typically developing (TD) controls; males with PM alleles (3.4 to 23.6 years); and males with FM only alleles and PM/FM mosaic alleles (1.89 to 43.17 years). Control children had no family history of developmental delay and their mothers had CGG size < 44.

Methylation analysis using Methylation-Specific Quantitative Melt Analysis (MS-QMA) identified methylated alleles in both brothers in multiple tissues (Figure 3A,B) that were not present in 17 control males and 14 PM males co-run with these samples. However, the methylated peaks of ~5% in both brothers did overlap with those found in 41 FM only males and 18 typical PM/FM mosaics also co-run with the B1 and B2 samples (Supplemental Figure S2). The levels observed in both brothers were also consistent with those of the spiking reference sample, which contained 6% methylated FM and 94% unmethylated normal size male DNA (Figure 3C). Presence of the unmethylated expanded alleles in the majority of cells was consistent with *FMR1* mRNA analyses in blood for both brothers (Figure 4), where the levels in both brothers were ~5 fold higher than the levels observed in male and female controls. Interestingly, of the 13 PM male reference samples (most children or adolescents), only one had analogous *FMR1* mRNA levels. None of the FM only or PM/FM mosaic males had mRNA levels remotely close to those observed in the brothers, with the C1 PM (66 CGGs) cousin having mRNA levels just above the control range. 

### 2.4. Neurodevelopmental Outcomes at the Time of Recruitment

In B1, the cognitive assessment as part of the FREE FX study highlighted receptive language difficulties, which greatly influenced his performance in the IQ test. He showed significant variability in his cognitive abilities. His verbal skills were a cognitive weakness, reflected in his verbal IQ (VIQ) score of 72 falling in the borderline range. His overall nonverbal problem-solving skills were in the low average range, with a performance IQ (PIQ) score of 84. His processing speed index (PSI) score was 71, which fell in the borderline range. His overall intellectual functioning, as reflected in the Full Scale IQ (FSIQ), was 77. Based on the Autism Diagnostic Observation Schedule Second Edition (ADOS-2) assessment, B1 met the cut-off for autism spectrum, with an overall ADOS-2 calibrated severity score (CSS) of 4. His Social Affect (SA) CSS was 3 (non-spectrum range), whilst his Restricted and Repetitive Behavior (RRB) CSS was 6 (autism range). B1’s scores on the Child Behavior Checklist (CBCL) were in the normal range for the Total Problem and Externalizing problem scales, but fell in the borderline range for the Internalizing problems scale. Moreover, the Pervasive Developmental Problems subscale score fell in the clinical range and the Withdrawn subscale score fell in the borderline range. At the time of the research assessment, B1 was enrolled in a small mainstream primary school with additional learning support. He was reported to play alongside other children and although still shy, he did not have any major challenging behaviours. He had no seizures and was taking 2 mg/day of sertraline.

During assessment, as part of the FREE FX study, B2 predominantly used non-word vocalizations and jabbering; although on a number of occasions, he used single words and word approximations. Moreover, his formal developmental assessment with the Mullen Scales of Early Learning (MSEL) showed that his receptive and expressive language skills were respectively similar to that expected of an infant aged 10 and 17 months old, a marked discrepancy from his chronological age (31 months). He did not obtain a valid score (T ≥ 20) on the receptive language subdomain of the MSEL; a further indication of the significant impairment in this area. B2′s ratio VIQ [36] was 44. In contrast, his performance in tasks assessing visual reception and fine motor skills was much better, obtaining age equivalents of 25 and 20 months, respectively, and a ratio non-verbal IQ (NVIQ) of 73. His overall MSEL Early Learning Composite score (using a default minimum T score of 20 for his Receptive Language domain) was 56, more than three standard deviations below the mean score (mean = 100; SD = 15) of similar aged peers, indicating a significant developmental delay. B2′s difficulties with social communication, his engagement in sensory seeking and repetitive behaviours, and restricted interests observed during the ADOS-2 assessment led to an ADOS-2 classification of autism, with an overall ADOS CSS of 7. His SA CSS score of 5 fell in the autism spectrum range, whereas his RRB CSS of 10 fell in the autism range, the highest severity level for this domain for children with ASD of his age and verbal abilities. B2′s CBCL Total, Internalizing, and Externalizing problem scales scores were all in the normal range. However, his score in the Withdrawn and Pervasive Developmental Problems subscales fell in the clinical range. His mother reported significant concerns regarding his very limited speech and social anxiety. 

## 3. Discussion 

This study describes two young brothers with expanded *FMR1* alleles, who were ‘higher functioning’ based on intellectual functioning assessments compared to a typical FXS cohort reported in previous studies [6]. Specifically, B1 had a PIQ score three standard deviations above the mean of the FREE FX typical FXS male cohort, while B2 had a non-verbal (NVIQ) score that was nearly two standard deviations above the same mean. Regarding VIQ, B1′s score was 1 SD greater than the FXS group mean, while B2 fell within 1 SD of the typical FXS cases on this measure. B2′s greater difficulties with expressive and receptive language skills may be closely linked to his more severe autism phenotype. In contrast, B1 presented mild autism features, though still met the ADOS-2 cut-off for autism spectrum. Based on methylation-sensitive Southern blot analysis of blood DNA, both brothers have been described as having UFM alleles approaching 200 CGGs in blood. Previous studies have described adults with similar Southern blot profiles, explaining their higher intellectual functioning through the *FMR1* promoter being completely unmethylated and expressing *FMR1* FM mRNA and FMRP [32,33,34]. These adult males with unmethylated alleles (FM and PM/FM mosaic) and incomplete silencing of *FMR1* mRNA from expanded FM alleles are at risk of FXTAS, based on clinical assessments and magnetic resonance imaging (MRI) features [30,37,38]. In these studies, it was hypothesized that expression of the UFM RNA is a contributor to the neurodegenerative features observed in some adults, through the same RNA toxicity mechanism as in PM-related disorders. However, the interplay between intellectual functioning and autism severity in the pediatric setting has not previously been characterized in individuals with UFM alleles. 

In this study, additional molecular analyses with more sensitive techniques, such as MS-QMA and AmplideX PCR, indicate that a small proportion of cells in blood and other tissues have methylated PM and FM alleles that were not detected by Southern blot analysis. These findings are partly consistent with a previous study examining tissue heterogeneity in *FMR1* methylation and CGG size post-mortem, in a 79-year-old ‘high-functioning’ male with unmethylated PM and FM alleles detected in blood by Southern blot analysis [34]. In this male, a complete unmethylated PM/FM allele smear was found in blood by Southern blot analysis; however, in multiple other tissues, including the parietal lobe, methylated FM alleles were detected. Together, this suggests that the detection of methylated alleles varies with the tissue tested and the analytical sensitivity of the technique used. With the addition of more sensitive technologies to complement Southern blot analysis, the UFM classification in some of these cases is likely to be replaced with the term ‘low-level methylation mosaicism’. Moreover, these males with low-level somatic mosaicism may not be as uncommon as previously thought. One reason for this may be that these unmethylated alleles are somatically unstable, with a significant proportion of these cases that have retraction down to a normal repeat size currently not being detected by standard testing [26]. 

It is also important to note that based on CGG sizing and methylation analysis of multiple tissues, B1 was found to have multiple methylated alleles in the PM range in a small proportion of cells (e.g., 170 CGG repeats in blood, but 140 and 156 CGG alleles in fibroblasts). This is consistent with an earlier study [26] that also reported a male with a methylated PM allele of 110 CGG repeats, in the absence of an FM, with a FSIQ value of 46. Together, this suggests that as part of somatic retraction below an FM allele size, some alleles may remain methylated in a proportion of cells, and may not be fully functional, with this proportion and CGG sizing differing between tissues. For the two brothers, however, the RNA toxicity, rather than FMRP deficiency, is likely to be the primary factor contributing to the autism phenotype. This is consistent with FM RNA levels being ~5 fold greater than in the reference sample of typically developing controls, PM/FM mosaic and FM only males affected with FXS, and the PM cousin (C1) with 66 CGGs. This is also consistent with our earlier study showing that FM males with incomplete silencing of *FMR1* had more severe autism features compared to the FM group where *FMR1* mRNA could not be detected [6].

This study also detected a potential sequence variant in the *FMR1* promoter overlapping with the 3′ MCA FastFraX primer binding site at the 3′ end of the CGG repeat. While the functional and clinical significance of this potential variant is uncertain, it did not inhibit *FMR1* transcription in both brothers. Similarly, previous studies have reported a sequence variant located next to the CGG repeat in FXS individuals displaying somatic retractions [30]. The loss of primer binding sites that may be associated with somatic retraction is an important limitation of all standard and long-range PCR-based methods, including AmplideX and FastFraX, as it may lead to expanded alleles being missed or not amplified, as demonstrated by the 3′ MCA assay for both brothers in this study, and for AmplideX previously [30]. 

In summary, the study reports two brothers with low-level methylation mosaicism not detected by standard testing in blood, who may be mistaken for males with rare UFM alleles, when based alone on Southern blot analysis of blood DNA. Moreover, in both brothers, *FMR1* mRNA levels were increased ~5-fold compared to typical developing controls, and significantly above the levels reported from PM, FM only, and PM/FM mosaic male cohorts from a previous study [6]. The abnormally elevated levels of *FMR1* mRNA in the two brothers may have led to *FMR1* mRNA-related cellular “toxicity”. It is plausible that an FXS patho-mechanism exists whereby active unmethylated FM and/or PM alleles lead to the expression of toxic expanded mRNA in some cells, in conjunction with possible reduced *FMR1* mRNA and FMRP levels in other cells with *FMR1* methylation detected by MS-QMA. A combination of these two mechanisms in different cells may contribute to the brothers’ neurodevelopmental problems, including the elevated ASD symptoms. This hypothesis is also in line with the findings reported by Baker et al. [6] showing that males with FM-only CGG expansions (aged < 19 years), who expressed FM *FMR1* mRNA, had significantly more severe ASD symptoms measured with the ADOS-2 compared to males with FM-only alleles who had completely silenced *FMR1*. The main limitations of this study are that the FMRP levels in blood and direct sequencing of the 3′ MCA binding site, anchored at the 5′ end of the CGG repeat, suspected to be modified in both brothers, have not been tested and performed. Future studies will address these limitations to shed light on the effect of the potential 3′ MCA variant (to be confirmed through DNA sequencing) on FMRP structure and function, and possibly further explain the phenotype presented by these two young children.

## 4. Materials and Methods 

### 4.1. Ethics Approval and Consent 

All aspects of this study have received ethical approval by The Royal Children’s Hospital Human Research Ethics Committee (Single Site Reference numbers: HREC 34227, HREC 33066—Multi site HREC Reference Number: HREC/13/RCHM/24, approved on 24 May 2013).

### 4.2. Recruitment and Assessments

Affected family members include two brothers, B1, age 5.6 years, and B2, age 2.7 years, and their cousin C1, age 3.4 years. All were recruited through their clinical geneticist into the FREE FX study [6]. As part of the FREE FX study, the brothers’ neurodevelopmental outcomes were evaluated through a medical and developmental history questionnaire prepared ad hoc for this study, the ADOS-2 (Module 2 for B1 and Module 1 for B2) [39], the Wechsler Preschool and Primary Scale of Intelligence–Third Edition (WPPSI-III) (Australian) (B1) [40], MSEL (B2) [41], and CBCL [42]. Both brothers’ and their cousin’s buccal epithelial cells, saliva, and venous blood samples were collected at the time of their participation in the study. A skin biopsy for genetic testing on fibroblasts was performed on B1, as part of his clinical care. Molecular data for positive and negative control cohorts used as reference data in this study were analysed previously [6,43,44] and from a newly recruited control and PM participants as part of the FREE FX study.

### 4.3. Sample Processing

Up to four BEC samples were collected per participant using the Master Amp Buccal Swab Brush kit (Epicentre Technologies, Madison, WI, USA), as previously described [45]. A single saliva sample was collected for each participant using the Oragene^®^ DNA Self-Collection Kit (DNA Genotek, Global) and was processed as per the manufacturer’s instructions. As part of a clinical genetic follow-up, and not as part of his involvement in the FREE FX study, a skin biopsy was performed for B1 to obtain fibroblasts. The DNA extracts were evaluated using a NanoDrop 2000 spectrophotometer (Thermo Fisher Scientific, Foster City, CA, USA). Ten ml of venous blood, from B1 and B2 and from two control male participants (aged 7.6 and 8.1 years, respectively), was used for peripheral blood mononuclear cell (PBMC) isolation using Ficoll gradient separation [46]. Isolated PBMCs were used for RNA extraction (RNeasy kit; Qiagen Inc., Hilden Germany) for gene expression analyses [46]. Total RNA extraction, purification, and reverse transcription were performed as previously described [47].

### 4.4. Methylation Specific-Quantitative Melt Analysis (MS-QMA) 

DNA samples were extracted from BEC, saliva, venous blood, and fibroblasts, and transferred into 96-well plates to be treated with sodium bisulphite. An EZ DNA Methylation-Gold^TM^ kit (Zymo research, Irvine, CA, USA) was used to bisulphite convert each sample in two separate reactions, with each conversion analysed in duplicate reactions. FREE2 DNA methylation analysis was performed using MS-QMA, as previously described [43]. Specifically, ninety-six samples were bisulfite converted at a time (3 controls and 93 unknown samples per plate) and were serially diluted four times post-conversion. These included positive and negative control cohorts used as reference data in this study, as well as samples in question from B1, B2, and C1. The bisulfite converted DNA was then transferred into a 384 well format for real-time PCR analysis utilizing MeltDoctor™ high-resolution melt reagents in 10 µL reactions, as per the manufacturer’s instructions (Life technologies, Foster City, CA, USA). A unique primer set was used for real-time PCR that targets specific CpG sites within the Fragile X-Related Epigenetic Element 2 (FREE2) region, at the *FMR1* exon1/intron 1 boundary [43]. The annealing temperature for the thermal cycling protocol was 650 °C for 40 cycles. The ViiA™ 7 Real-Time PCR System (Life technologies, Foster City, CA, USA) was then used to quantify the DNA concentration of the unknown samples using the relative standard curve method post bisulfite conversion, by measuring the rate of dye incorporation into double stranded DNA. To progress to the next stage of the MS-QMA analysis, the unknown samples had to be within this dynamic linear range. 

The products from a methylated and unmethylated FREE2 sequence were then separated into single strands in the temperature range of 74 and 82 °C as part of the high-resolution melt step that followed the real-time PCR in a close tube format. The HRM Software Module for ViiA™ 7 System was then used to plot the rate of PCR product separation to single strands, with the difference in fluorescence converted to Aligned Fluorescence Units (AFU).

The AFU conversion to the methylation percentage was performed at 78 °C, and all of the above quality control steps, were analysed simultaneously for 384 reactions at a time using Q-MAX software (Curve Tomorrow, Melbourne, Australia), developed to automate the process. 

This software utilizes a custom-designed computer algorithm to simultaneously perform multiple quality control checks to determine DNA concentrations and quality post-bisulfite conversion using raw RT-PCR and high-resolution melt data for all dilutions from each bisulfite reaction. The high-resolution melt data for those sample dilutions outside the QC ranges were discarded from the quantitative methylation analysis by Q-MAX software, and were not used for the final aggregate methylation ratio calculation. The high-resolution melt profiles discarded from quantitative assessments by Q-MAX, however, were used for visual assessments for the presence or absence of abnormal methylation compared to control melt curves (Figure 3). Males were considered to have positive MS-QMA results if they had >2% methylation derived through Q-MAX and/or one of more high-resolution melt curve profiles that had derivative high-resolution melt plots with the presence of a peak originating from methylated alleles, at the same melting temperature as in FXS male controls (Figure 3). 

### 4.5. Methylation Analysis and CGG Sizing Using Southern Blot and AmplideX PCR 

Methylation-sensitive Southern blot analysis of the *NruI* restriction site within the *FMR1* CpG island was performed for the two brothers’ venous blood DNA samples, using a fully validated methylation-sensitive Southern blot procedure with appropriate normal and abnormal controls, as described previously [48]. The *FMR1* CGG repeat size was assessed using a fully validated PCR assay with a precision of +/− one triplet repeat across the normal and grey zone ranges, performed using a fragment analyser (MegaBACE, GE Healthcare, Chicago, Illinois, IL, USA), with the upper limit of detection of 170 repeats, as described previously [49]. CGG sizing using both brothers’ BEC, saliva, venous blood, and fibroblast (only B2) DNA was also performed using the AmplideX^®^™ *FMR1* PCR Kit as per the manufacturer’s instructions [50] (Asuragen, Austin, TX, USA). The *FMR1* CGG size in DNA extracted from venous blood from the two brothers was further investigated by Southern blot testing at the Victorian Clinical Genetics Services (VCGS) (without the use of methylation sensitive restriction enzymes), as described previously [48].

### 4.6. Melting Curve Analysis (MCA) to Determine the Presence of Expanded FMR1 Alleles

Five prime and 3′ MCA high-resolution melt analyses were also undertaken in the BEC, saliva, and venous blood DNA of both brothers, in the fibroblasts DNA of B1, and in the venous blood DNA of C1, as per the manufacturer’s instructions for the FastFraX commerical kit (Biofactory, Singapore) [35]. Specifically, 5′ and 3′ PCR reactions were performed for each sample, with each assay having one of the primers anchored at one end of the CGG repeat. Each assay contained 5 units of HotStarTaq DNA polymerase (Qiagen), 2.5× Q-Solution (Qiagen), 1× of the supplied PCR buffer (Qiagen), 0.1× SYBR Green I nucleic acid dye (Roche Applied Science, Upper Bavaria, Germany), and 50 ng genomic DNA. The 5′ PCR used a deoxynucleoside triphosphate mix consisting of 0.2 mmol/L each of dATP, dTTP, and dCTP, and 0.1 mmol/L each of 7-deaza-2′-dGTP (7-deaza-dGTP) and dGTP (Roche Molecular Diagnostics, Upper Bavaria, Germany). The 3′-PCR used a deoxynucleoside triphosphate mix consisting of 0.2 mmol/L each of dATP, dTTP, dCTP, and dGTP, and primer sequences and concentrations as previously described [35], with primer locations indicated in Figure 2A. The 5′ and 3′-PCR reactions were performed separately under identical thermocycling conditions in a ViiA™ 7 Real-Time PCR System (Life technologies, Foster City, CA). An initial denaturation step at 95 °C for 15 min was followed by 40 cycles of 99 °C for 2 min, 65 °C for 2 min, and 72 °C for 3 min, and then a final extension step at 72 °C for 10 min. PCR amplicons were then melted (after completion of the PCR program), consisting of denaturation at 95 °C for 1 min, a temperature-hold step at 60 °C for 1 min, and a temperature ramp from 60 °C to 95 °C at a rate of 0.01 °C/s. Reference male samples were co-run with the samples in question, including an FM (530 CGG), a PM (170 CGG), and a normal size (NS) control (30 CGG). Positive and negative calls were made based on the presence or absence of the difference in the profile fluorescence at the melting temperature threshold (85 °C for 5′ MCA; 90 °C for 3′MCA) compared to male reference samples. 

### 4.7. Sanger Sequencing

Sanger sequencing of the 3′ MCA *FMR1* exon 1 binding site inclusive of the ATG translation start site, and surrounding regions was performed at the Australian Genome Research Facility Ltd in blood of B1 and B2, and compared to 2 reference samples from typically developing controls co-run with these samples. The data was re-analysed at the Victorian Clinical Genetics Services (VCGS), using Mutation Surveyor® V4.0.9 software (SoftGenetics®, State College, PA, USA) to detect and report SNPs as previously described [51].

### 4.8. FMR1 mRNA Analysis

Gene expression analyses were performed using reverse transcription real-time quantitative PCR (RT-PCR) on a ViiaTM 7 System (Life Technologies, Global), with the relative standard curve method as described in Kraan et al. [52]. The mean 5’and 3′ *FMR1* mRNA levels were normalized to the mean of *EIF4A2* and *SDHA* mRNA levels used as internal controls, expressed in arbitrary units (a.u) [52]. The summary measure used for mRNA expression levels for each participant was calculated by averaging the four arbitrary unit outputs originating from the two separate cDNA reactions, with each of these analysed in two separate RT-PCR reactions, performed for each RNA sample [52,53]. 

## Supplementary Materials

The following are available online at https://www.mdpi.com/1422-0067/20/16/3907/s1.

## References

[1] Vissers L.E., Gilissen C., Veltman J.A. (2016). Genetic studies in intellectual disability and related disorders. Nat. Rev. Genet..

[2] Clifford S., Dissanayake C., Bui Q.M., Huggins R., Taylor A.K., Loesch D.Z. (2007). Autism spectrum phenotype in males and females with fragile X full mutation and premutation. J. Autism Dev. Disord..

[3] Hall S.S., Lightbody A.A., Reiss A.L. (2008). Compulsive, self-injurious, and autistic behavior in children and adolescents with fragile X syndrome. Am. J. Ment. Retard..

[4] Mazzocco M.M., Kates W.R., Baumgardner T.L., Freund L.S., Reiss A.L. (1997). Autistic behaviors among girls with fragile X syndrome. J. Autism Dev. Disord..

[5] Merenstein S.A., Sobesky W.E., Taylor A.K., Riddle J.E., Tran H.X., Hagerman R.J. (1996). Molecular-clinical correlations in males with an expanded *FMR1* mutation. Am. J. Med. Genet..

[6] Baker E.K., Arpone M., Aliaga S.M., Bretherton L., Kraan C.M., Bui M., Slater H.R., Ling L., Francis D., Hunter M.F. (2019). Incomplete silencing of full mutation alleles in males with fragile X syndrome is associated with autistic features. Mol. Autism..

[7] Kraan C.M., Godler D.E., Amor D.J. (2018). Epigenetics of fragile X syndrome and fragile X-related disorders. Dev. Med. Child Neurol..

[8] Heitz D., Rousseau F., Devys D., Saccone S., Abderrahim H., Le Paslier D., Cohen D., Vincent A., Toniolo D., Della Valle G. (1991). Isolation of sequences that span the fragile X and identification of a fragile X-related CpG island. Science.

[9] Oberle I., Rousseau F., Heitz D., Kretz C., Devys D., Hanauer A., Boue J., Bertheas M.F., Mandel J.L. (1991). Instability of a 550-base pair DNA segment and abnormal methylation in fragile X syndrome. Science.

[10] Pieretti M., Zhang F.P., Fu Y.H., Warren S.T., Oostra B.A., Caskey C.T., Nelson D.L. (1991). Absence of expression of the FMR-1 gene in fragile X syndrome. Cell.

[11] Rousseau F., Heitz D., Biancalana V., Blumenfeld S., Kretz C., Boué J., Tommerup N., Hagen C.V.D., DeLozier-Blanchet C., Croquette M.-F. (1991). Direct diagnosis by DNA analysis of the fragile X syndrome of mental retardation. N. Engl. J. Med..

[12] Sutcliffe J.S., Nelson D.L., Zhang F., Pieretti M., Caskey C.T., Saxe D., Warren S.T. (1992). DNA methylation represses FMR-1 transcription in fragile X syndrome. Hum. Mol. Genet..

[13] Devys D., Lutz Y., Rouyer N., Bellocq J.-P., Mandel J.-L. (1993). The FMR–1 protein is cytoplasmic, most abundant in neurons and appears normal in carriers of a fragile X premutation. Nat. Genet..

[14] Feng Y., Zhang F., Lokey L., Chastain J., Lakkis L., Eberhart D., Warren S. (1995). Translational suppression by trinucleotide repeat expansion at *FMR1*. Science.

[15] Pretto D., Yrigollen C.M., Tang H.T., Williamson J., Espinal G., Iwahashi C.K., Durbin-Johnson B., Hagerman R.J., Hagerman P.J., Tassone F. (2014). Clinical and molecular implications of mosaicism in *FMR1* full mutations. Front. Genet..

[16] Willemsen R., Smits A., Mohkamsing S., van Beerendonk H., de Haan A., de Vries B., van den Ouweland A., Sistermans E., Galjaard H., Oostra B.A. (1997). Rapid antibody test for diagnosing fragile X syndrome: A validation of the technique. Hum. Genet..

[17] Gothelf D., Furfaro Joyce A., Hoeft F., Eckert Mark A., Hall Scott S., O’Hara R., Erba Heather W., Ringel J., Hayashi Kiralee M., Patnaik S. (2007). Neuroanatomy of fragile X syndrome is associated with aberrant behavior and the fragile X mental retardation protein (FMRP). Ann. Neurol..

[18] Irwin S.A., Galvez R., Greenough W.T. (2000). Dendritic spine structural anomalies in fragile-X mental retardation syndrome. Cereb Cortex.

[19] Jeanne W.I., Greenough W.T. (1999). Synaptic synthesis of the fragile X protein: Possible involvement in synapse maturation and elimination. Am. J. Med. Genet..

[20] Pfeiffer B.E., Huber K.M. (2009). The state of synapses in fragile X syndrome. Neuroscientist.

[21] Sidorov M.S., Auerbach B.D., Bear M.F. (2013). Fragile X mental retardation protein and synaptic plasticity. Mol. Brain.

[22] Rodriguez-Revenga L., Madrigal I., Badenas C., Xuncla M., Jimenez L., Mila M. (2009). Premature ovarian failure and fragile X female premutation carriers: No evidence for a skewed X-chromosome inactivation pattern. Menopause.

[23] Sherman S.L. (2000). Premature ovarian failure in the fragile X syndrome. Am. J. Med. Genet..

[24] Nolin S.L., Glicksman A., Houck G.E., Brown W.T., Dobkin C.S. (1994). Mosaicism in fragile X affected males. Am. J. Med. Genet..

[25] Rousseau F., Heitz D., Tarleton J., MacPherson J., Malmgren H., Dahl N., Barnicoat A., Mathew C., Mornet E., Tejada I. (1994). A multicenter study on genotype-phenotype correlations in the fragile X syndrome, using direct diagnosis with probe StB12.3: The first 2,253 cases. Am. J. Hum. Genet..

[26] Aliaga S.M., Slater H.R., Francis D., Du Sart D., Li X., Amor D.J., Alliende A.M., Santa Maria L., Faundes V., Morales P. (2016). Identification of Males with Cryptic Fragile X Alleles by Methylation-Specific Quantitative Melt Analysis. Clin. Chem..

[27] Bonarrigo F.A., Russo S., Vizziello P., Menni F., Cogliati F., Giorgini V., Monti F., Milani D. (2014). Think about it: *FMR1* gene mosaicism. J. Child Neurol..

[28] Jiraanont P., Kumar M., Tang H.-T., Espinal G., Hagerman P.J., Hagerman R.J., Chutabhakdikul N., Tassone F. (2017). Size and methylation mosaicism in males with fragile X syndrome. Expert Rev. Mol. Diagn..

[29] Orrico A., Galli L., Dotti M.T., Plewnia K., Censini S., Federico A. (1998). Mosaicism for full mutation and normal-sized allele of the *FMR1* gene: A new case. Am. J. Med. Genet..

[30] Hwang T.Y., Aliaga S., Arpone M.V., Francis D., Li X., Chong B., Slater H.R., Rogers C., Bretherton L., Hunter M. (2016). Partially Methylated Alleles, Microdeletion and Tissue Mosaicism in a Fragile X Male with Tremor and Ataxia at 30 Years of Age: A Case Report. Am. J. Med. Genet..

[31] Roid G.H. (2003). Stanford-Binet Intelligence Scales-Fifth Edition (SB5) Examiner’s Manual.

[32] Hagerman R.J., Hull C.E., Safanda J.F., Carpenter I., Staley L.W., O’Connor R.A., Seydel C., Mazzocco M.M., Snow K., Thibodeau S.N. (1994). High functioning fragile X males: Demonstration of an unmethylated fully expanded FMR-1 mutation associated with protein expression. Am. J. Med. Genet..

[33] Basuta K., Schneider A., Gane L., Polussa J., Woodruff B., Pretto D., Hagerman R., Tassone F. (2015). High functioning male with fragile X syndrome and fragile X-associated tremor/ataxia syndrome. Am. J. Med. Genet. A.

[34] Taylor A.K., Tassone F., Dyer P.N., Hersch S.M., Harris J.B., Greenough W.T., Hagerman R.J. (1999). Tissue heterogeneity of the *FMR1* mutation in a high-functioning male with fragile X syndrome. Am. J. Med. Genet..

[35] Teo C.R., Law H.Y., Lee C.G., Chong S.S. (2012). Screening for CGG repeat expansion in the *FMR1* gene by melting curve analysis of combined 5′ and 3′ direct triplet-primed PCRs. Clin. Chem..

[36] Bishop S.L., Guthrie W., Coffing M., Lord C. (2011). Convergent validity of the Mullen Scales of Early Learning and the differential ability scales in children with autism spectrum disorders. Am. J. Intellect. Dev. Disabil..

[37] Santa Maria L., Pugin A., Alliende M., Aliaga S., Curotto B., Aravena T., Tang H.T., Mendoza-Morales G., Hagerman R., Tassone F. (2013). FXTAS in an unmethylated mosaic male with fragile X syndrome from Chile. Clin. Genet..

[38] Hwang Y.T., Dudding T., Aliaga S.M., Arpone M., Francis D., Li X., Slater H.R., Rogers C., Bretherton L., du Sart D. (2016). Molecular Inconsistencies in a Fragile X Male with Early Onset Ataxia. Genes.

[39] Lord C., Rutter M., DiLavore P., Risi S., Gotham K., Bishop S. (2012). Autism Diagnostic Observation Schedule, Second Edition: ADOS-2.

[40] Wechsler D. (2004). Wechsler Preschool & Primary Scale of Intelligence–Third Edition Australian Standardised Edition (WPPSI-III Australian).

[41] Mullen E.M. (1995). Mullen Scales of Early Learning: AGS Edition (MSEL:AGS).

[42] Achenbach T.M., Rescorla L.A. (2000). Manual for the ASEBA Preschool Forms & Profiles.

[43] Inaba Y., Schwartz C.E., Bui Q.M., Li X., Skinner C., Field M., Wotton T., Hagerman R.J., Francis D., Amor D.J. (2014). Early Detection of Fragile X Syndrome: Applications of a Novel Approach for Improved Quantitative Methylation Analysis in Venous Blood and Newborn Blood Spots. Clin. Chem..

[44] Godler D.E., Inaba Y., Schwartz C.E., Bui Q.M., Shi E.Z., Li X., Herlihy A.S., Skinner C., Hagerman R.J., Francis D. (2015). Detection of skewed X-chromosome inactivation in Fragile X syndrome and X chromosome aneuploidy using quantitative melt analysis. Expert Rev. Mol. Med..

[45] Arpone M., Baker E.K., Bretherton L., Bui M., Li X., Whitaker S., Dissanayake C., Cohen J., Hickerton C., Rogers C. (2018). Intragenic DNA methylation in buccal epithelial cells and intellectual functioning in a paediatric cohort of males with fragile X. Sci. Rep..

[46] Loesch D.Z., Godler D.E., Evans A., Bui Q.M., Gehling F., Kotschet K.E., Trost N., Storey E., Stimpson P., Kinsella G. (2011). Evidence for the toxicity of bidirectional transcripts and mitochondrial dysfunction in blood associated with small CGG expansions in the *FMR1* gene in patients with parkinsonism. Genet. Med..

[47] Godler D.E., Loesch D.Z., Huggins R., Gordon L., Slater H.R., Gehling F., Burgess T., Choo K.H. (2009). Improved methodology for assessment of mRNA levels in blood of patients with *FMR1* related disorders. BMC Clin. Pathol..

[48] Francis D., Burgess T., Mitchell J., Slater H. (2000). Identification of small FRAXA premutations. Mol. Diagn..

[49] Khaniani M.S., Kalitsis P., Burgess T., Slater H.R. (2008). An improved Diagnostic PCR Assay for identification of Cryptic Heterozygosity for CGG Triplet Repeat Alleles in the Fragile X Gene (*FMR1*). Mol. Cytogenet..

[50] Chen L., Hadd A.G., Sah S., Houghton J.F., Filipovic-Sadic S., Zhang W., Hagerman P.J., Tassone F., Latham G.J. (2011). High-resolution methylation polymerase chain reaction for fragile X analysis: Evidence for novel *FMR1* methylation patterns undetected in Southern blot analyses. Genet. Med..

[51] Weckx S., Del-Favero J., Rademakers R., Claes L., Cruts M., De Jonghe P., Van Broeckhoven C., De Rijk P. (2005). novoSNP, a novel computational tool for sequence variation discovery. Genome Res..

[52] Kraan C.M., Cornish K.M., Bui Q.M., Li X., Slater H.R., Godler D.E. (2016). β-glucuronidase mRNA levels are correlated with gait and working memory in premutation females: Understanding the role of FMR1 premutation alleles.. Sci. Rep..

[53] Kraan C.M., Cornish K.M., Bui Q.M., Li X., Slater H.R., Godler D.E. (2018). beta-glucuronidase use as a single internal control gene may confound analysis in *FMR1* mRNA toxicity studies. PLoS ONE.

