# Supplementary material for: Significantly Elevated FMR1 mRNA and Mosaicism for Methylated Premutation and Full Mutation Alleles in Two Brothers with Autism Features Referred for Fragile X Testing
Source: Int J Mol Sci. 2019 Aug 11;20(16):3907. doi: 10.3390/ijms20163907 (PMC6721168; doi:10.3390/ijms20163907)
Supplement: Supplementary file 1 [file ijms-20-03907-s001.pdf]

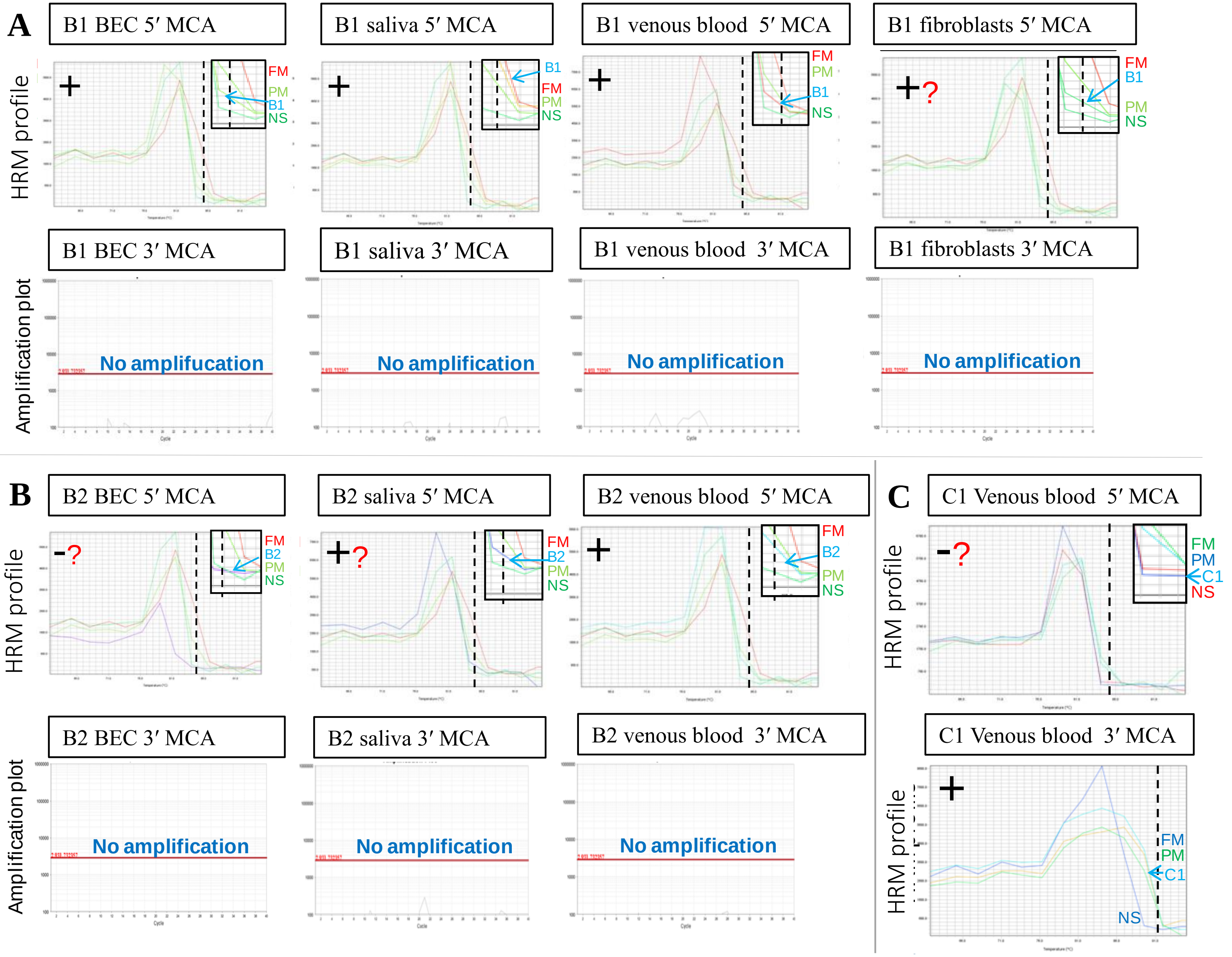

Supplemental Figure 1. FastFraX triplet-primed PCR results using 5'MCA and 3'MCA assays for B1 **(A)** in Buccal Epithelial Cell (BEC), saliva, venous blood and fibroblasts DNA, for B2 **(B)** in BEC, saliva, and venous blood, and for C1 **(C)** in venous blood. *Note*: Reference male samples co-run with the samples in question included a FM (530 CGG), a PM (170 CGG) and a normal size (NS) control (30 CGG). ? Indicates uncertain calls where the difference in the profile fluorescence at the melting temperature threshold (85°C for 5'MCA; 90 °C for 5'MCA) is insufficient to differentiate the sample in question from NS. (+) indicates positive call; (-) indicates negative call. It appears that 5'MCA assay missed PM allele calls in B2 BEC and C1 venous blood.

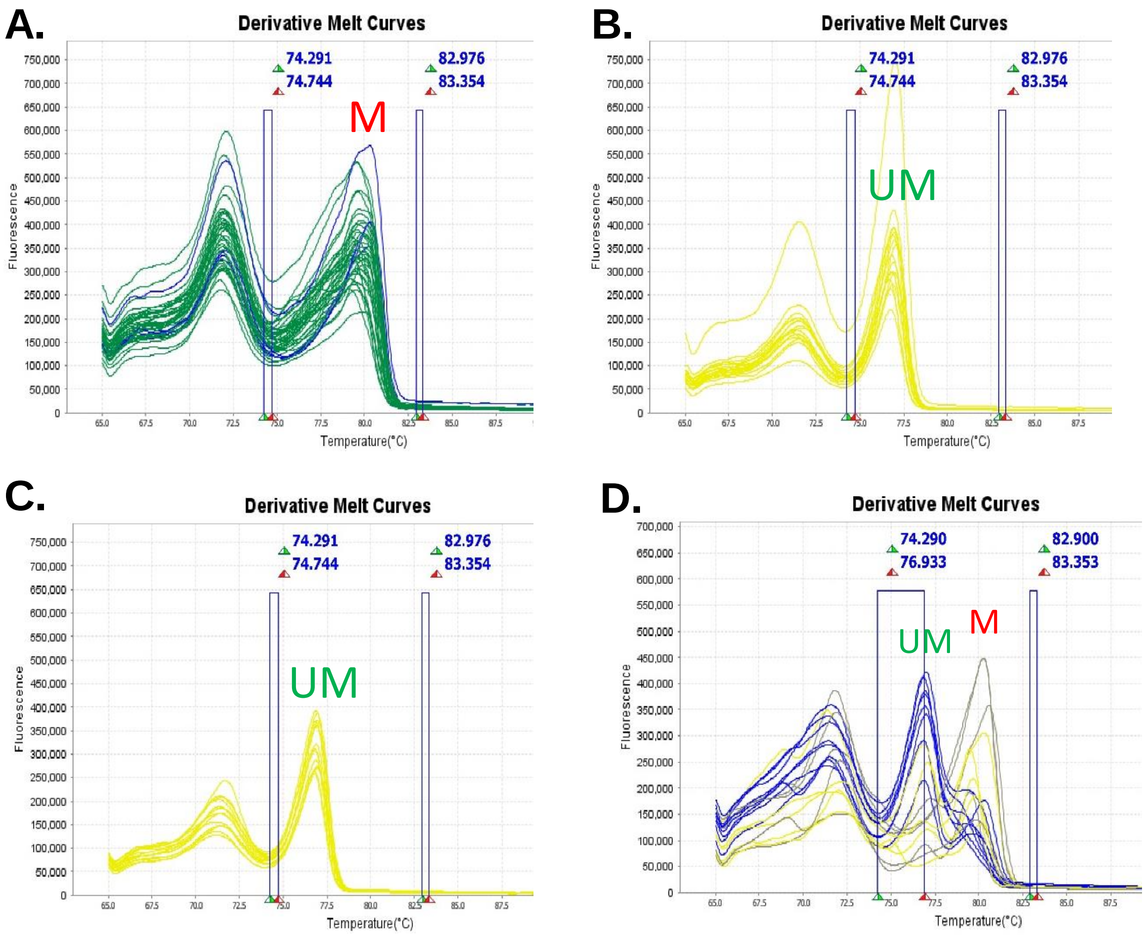

Supplemental Figure 2. Derivative curve high resolution melt profiles from the FREE2 MS-QMA assay between in blood DNA reference samples from (A) 41 FM only; (B) 14 PM; (C) 17 controls; and (D) 18 PM/FM mosaic males co-run with the samples in question. Note: M = methylated alleles; UM = unmethylated alleles.

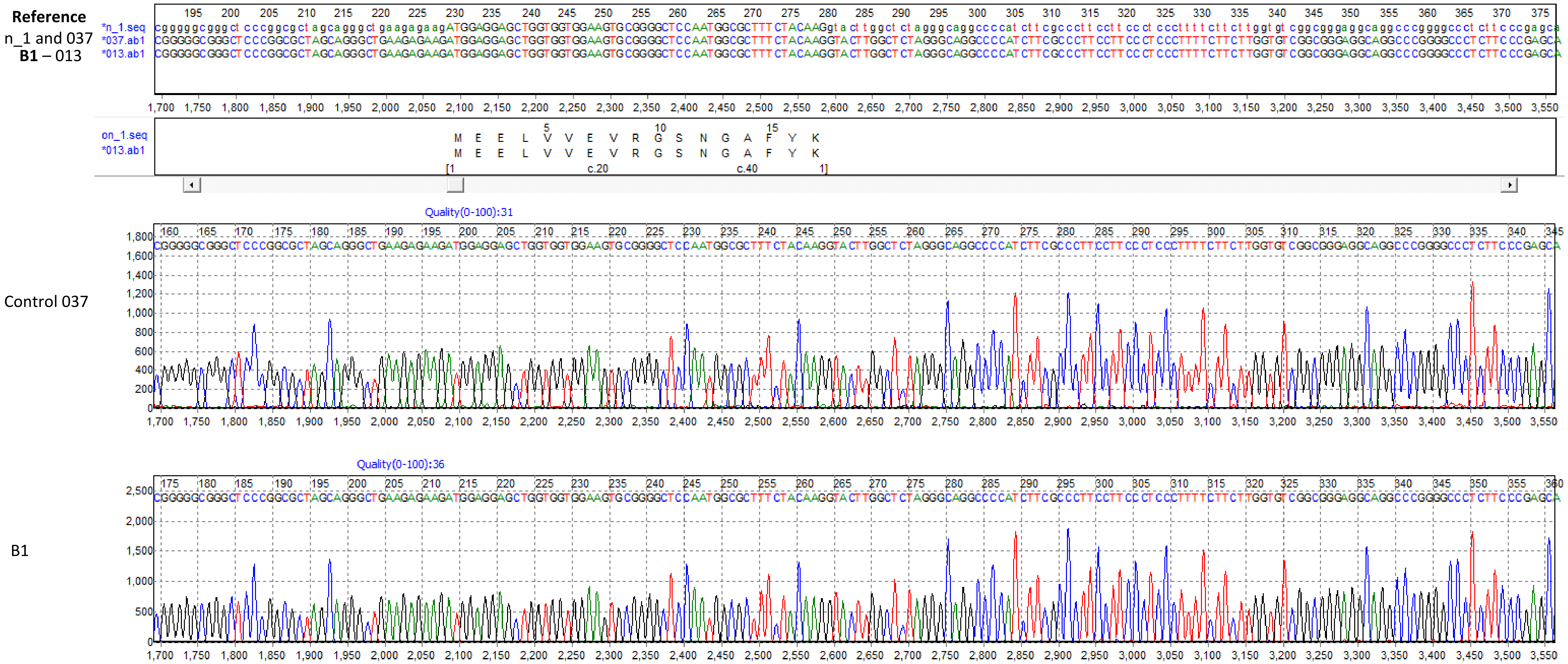

Supplemental Figure 3. Sanger sequencing within of the 3’MCA *FMR1* exon 1 binding site inclusive of the ATG translation start site, and surrounding regions detected no sequence variants in blood of B2 (013.ab1 sequence) as compared to 2 reference samples (Controls n_1.seq and 037.ab1) from typically developing controls.

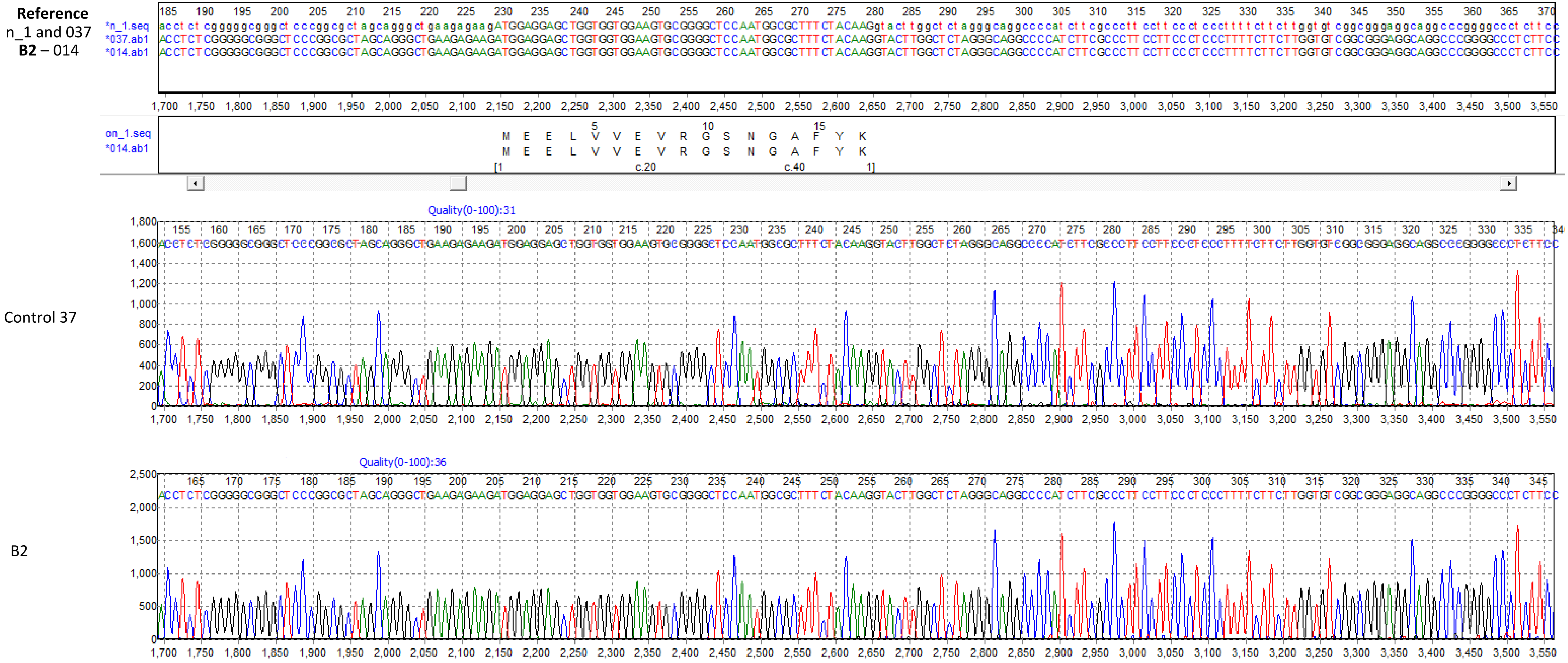

Supplemental Figure 4. Sanger sequencing within of the 3'MCA *FMR1* exon 1 binding site inclusive of the ATG translation start site, and surrounding regions detected no sequence variants in blood of B2 (014.ab1 sequence) as compared to 2 reference samples (Controls n_1.seq and 037.ab1) from typically developing controls.
